# Gastroscopic Evaluation of Gastric Mucosa in Horses Treated With Flunixin Meglumine or High‐Dose Firocoxib: A Descriptive Pilot Study

**DOI:** 10.1002/vms3.71061

**Published:** 2026-07-08

**Authors:** Renato Abrantes de Oliveira, Alexandre da Silva Correa, Francisco Décio de Oliveira Monteiro, Pedro Paulo Maia Teixeira

**Affiliations:** ^1^ Veterinary Medicine Institute Federal University of Pará Castanhal Pará Brazil; ^2^ Federal Institute of Tocantins (IFTO), Campus Araguatins Araguatins Tocantins Brazil

**Keywords:** equine, firocoxib, flunixin meglumine, gastric ulcer, gastroscopy, histopathology, NSAID

## Abstract

**Background:**

Equine Gastric Ulcer Syndrome (EGUS) is common in horses, and nonsteroidal anti‐inflammatory drugs (NSAIDs) are a known risk factor for gastrointestinal ulceration. Although COX‐2 selective NSAIDs such as firocoxib theoretically offer a better safety profile, endoscopic evidence for their mucosal safety at high off‐label doses is lacking. Objectives: To compare the effects of label‐dose flunixin meglumine and high‐dose firocoxib on the gastric mucosa of a small selected group of horses using serial gastroscopy and histopathology in a descriptive pilot study.

**Methods:**

Twenty mixed‐breed horses were allocated non‐randomly to control (*n* = 10), flunixin meglumine (1.1 mg/kg IM, *n* = 5), or firocoxib (1.0 mg/kg PO, *n* = 5) groups, treated once daily for 5 days. Gastroscopic evaluation and histopathological analysis were performed before (Day 1) and after treatment (Day 6).

**Results:**

No glandular disease was observed. Low prevalence of pre‐existing squamous lesions was found in all groups without progression after treatment. Histopathological scores revealed baseline incomparability, with marked cellular infiltrate present only in the flunixin group at Day 1 (60%). Within‐group analyses showed no significant changes between time points (*p* > 0.05). The use of NSAIDs effectively resolved lameness.

**Conclusions:**

In this small descriptive pilot study, limited by non‐randomised allocation and small sample size, the study found no detectable short‐term gastric mucosal injury associated with high‐dose firocoxib within the limits of this study. Due to the non‐randomised design, no causal conclusions can be drawn. These hypothesis‐generating findings require confirmation in adequately powered randomised trials.

## Graphical abstract

In this descriptive pilot study, short‐term (5‐day) high‐dose firocoxib (1.0 mg/kg) was not associated with detectable gastric mucosal injury in horses. Due to non‐randomised design and small sample size (*n* = 5/group), no causal conclusions can be drawn. Findings are hypothesis‐generating and require confirmation in adequately powered trials.

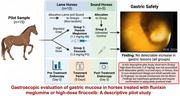

## 1 Introduction

Equine Gastric Ulcer Syndrome (EGUS) is the most common disease of the equine stomach, representing a clinical and welfare concern in animal (Spanton 2024; Vokes et al. 2023). EGUS, encompassing both squamous (ESGD) and glandular (EGGD) mucosal disease, can be diagnosed by gastroscopy, the gold standard for definitive diagnosis and monitoring of this pathology (Sykes et al. 2015; Van den Boom 2022). Despite its high prevalence, diagnosing EGUS can be challenging as clinical signs are often vague or absent, necessitating a reliance on direct endoscopic evaluation when risk factors are present (Ferlini Agne et al. 2023; Frippiat et al. 2025). This diagnostic reality underscores the importance of understanding iatrogenic risk factors that clinicians can control, particularly pharmacologic therapies.

Non‐steroidal anti‐inflammatory drugs (NSAIDs) are indispensable in equine practice for managing pain, inflammation, and fever (Duz et al. 2019; Mercer et al. 2023). However, non‐selective inhibition of cyclooxygenase enzymes is a well‐established risk factor for gastrointestinal toxicity, including ulceration (Davis 2017; Flood and Stewart 2022). Flunixin meglumine is among the most frequently prescribed NSAIDs for conditions such as colic and musculoskeletal pain, placing a large population of horses at risk (Ziegler et al. 2019). The quest for effective analgesia with a reduced side‐effect profile has driven the adoption of COX‐2 selective NSAIDs like firocoxib, which are theorised to spare the gastroprotective prostaglandins synthesised via COX‐1 (Cook and Blikslager 2015).

Despite their theoretical advantage, direct gastroscopic evidence supporting the mucosal safety profile of firocoxib, particularly at higher doses, remains limited (Araújo et al. 2023). Comparative studies between flunixin meglumine and firocoxib have focused on clinical efficacy, pharmacokinetics, and specific outcomes like post‐operative recovery or endotoxemia management, but direct, blinded gastroscopic evaluations are less common (Cook et al. 2009; Duz et al. 2015). Furthermore, existing safety assessments often utilise standard label doses, leaving a gap in knowledge regarding the gastric effects of supratherapeutic dosing, which may occur in practice or be required for certain conditions (Bishop et al. 2023). This gap is critical for clinical decision‐making, where the safety of a selective NSAID must be balanced with anti‐inflammatory effects.

Therefore, this study was designed to provide preliminary clinically relevant evidence by performing a serial gastroscopic and histopathological evaluation of the gastric mucosa in horses treated with a label dose of flunixin meglumine and a high dose of firocoxib over a period of 5 days. By employing the definitive diagnostic tool (gastroscopy) supported by histopathology, this investigation aims to directly visualise and characterise any treatment‐associated mucosal changes (Camacho‐Luna et al. 2018; Van den Boom 2022). The findings will contribute essential data to equine clinical practice, providing veterinarians with evidence‐based guidance on the safety profile of two pivotal NSAIDs for therapeutic treatment of pain and inflammation.

## 2 Materials and Methods

### 2.1 Ethical Approval and Study Design

This was a descriptive, non‐randomised, prospective pilot study. The study was carried out according to the recommendations of the Brazilian National Council for Animal Experimentation Control (CONCEA) and was approved by the Ethics Committee for the Use of Animals of the Federal University of Pará under protocol number 8805210920 on 15 March 2019. This prospective, controlled clinical study was carried out in a population of horses of the military police battalion. The non‐randomised design precludes causal inference; findings are descriptive and hypothesis‐generating.

### 2.2 Animals and Management

Twenty geldings mixed‐breed (Creole/Thoroughbred), with a mean age of 12.0 years (±2.8 years SD; range: 8–16 years) and a mean body weight of 490 kg (±35 kg SD; range: 430–540 kg), were used in this study. All animals belonged to the Mounted Police Regiment. The horses were housed in individual confined pens (20 m^2^) without pasture access. For more than five years prior to the study, they received a standardised diet consisting of a commercial concentrate (AF 17% Equine Feed, 3 kg per animal three times daily) containing incorporated green forage (alfalfa hay – *Medicago sativa*) at a proportion of approximately 40% of the total ration. In addition to the alfalfa incorporated into the concentrate, the horses had free access to Tifton 85 hay (*Cynodon* spp.) in hay racks within their pens, which was replenished twice daily. This hay provided long‐stem roughage to support gastric health and normal digestive function. Water was available ad libitum. The alfalfa hay incorporated into the concentrate provided approximately 2.4 kg per animal per day, while the Tifton 85 hay was offered ad libitum, with average daily consumption estimated at 5–7 kg per animal.

Lameness was diagnosed and graded by a single experienced veterinarian using a standardised protocol. All horses underwent a baseline lameness evaluation on Day 1 prior to treatment allocation. The evaluation consisted of:

1. Visual inspection of the horse at rest (assessment of posture, limb carriage, and hoof placement);
2. Lameness examination in‐hand on a straight line (hard surface, 50 m) at walk and trot;
3. Lameness examination on a circle (10‐m diameter) on both left and right reins at trot;
4. Flexion tests (distal and proximal) followed by trot‐off assessment

Horses included in the treatment groups presented with lameness localised to the distal limb, all of which had resolved or were under control by Day 6 following NSAID treatment. No horses required additional veterinary intervention beyond the study protocol.

#### 2.2.1 Study Timeline and Inclusion

All horses were included in the study concurrently over a single 6‐day period. The study was conducted during the month of May 2019 (dry season in the region). On Day 1, all 20 horses underwent baseline clinical examination, anamnesis, lameness evaluation, and gastroscopy with biopsy collection. Horses meeting the inclusion criteria (no prior NSAID use within 60 days, no history of colic or gastrointestinal disease) were enrolled. The 10 lame horses were then assigned to the two treatment groups (Gflux, *n* = 5; Gfiro, *n* = 5), while the 10 sound horses formed the control group (Gcont, *n* = 10). Treatments were administered once daily for five consecutive days (Days 1–5). On Day 6, all horses underwent repeat clinical examination and gastroscopy with biopsy collection. This concurrent design minimised potential confounding from seasonal or environmental variations.

### 2.3 Experimental Groups and Drug Administration

The horses were subjected to a complete clinical examination and anamnesis. Due to the operational constraints of the military police battalion from which the horses were sourced, random allocation to groups was not feasible, and this study is therefore descriptive and hypothesis‐generating rather than confirmatory. No causal inferences can be drawn from the comparisons presented. Instead, a pragmatic allocation strategy was employed based on clinical presentation.

No formal matching of groups was performed due to the limited number of lame horses available during the study period. However, baseline comparability was assessed for the following parameters: age, body weight, body condition score, physiological parameters (heart rate, respiratory rate, rectal temperature, capillary refill time), historical risk factors (colic, trauma, prior NSAID use), and diet. As reported in Section 3.4 and Table 1, these parameters did not differ significantly between groups at baseline. Nevertheless, the non‐randomised design cannot exclude confounding by unmeasured variables (e.g., pain‐associated stress, systemic inflammation, behavioural factors) that may influence gastric mucosal status independently of treatment.

Ten animals presenting locomotor problems (e.g., lameness) and with no history of colic, gastrointestinal disease, or NSAID administration in the preceding 6 months were assigned to treatment groups. The 10 clinically sound animals, also free from any historical or clinical evidence of gastrointestinal disease or recent NSAID exposure, formed the control group. Thus, three groups were established:

Control Group (Gcont, *n* = 10): Comprised 10 clinically sound animals with no evidence of lameness or locomotor abnormalities on clinical examination, and with no history of colic, gastrointestinal disease, or NSAID administration in the preceding 6 months. These horses received no anti‐inflammatory therapy but were administered a sterile solution of 0.9% sodium chloride (NaCl) in purified water intramuscularly (IM) once daily for 5 days to control for handling and injection procedures associated with the flunixin meglumine group.

Flunixin Meglumine Group (Gflux, *n* = 5): Comprised five animals presenting with locomotor problems (e.g., lameness) on clinical examination, with no history of colic, gastrointestinal disease, or NSAID administration in the preceding 6 months. These horses were treated with label‐dose flunixin meglumine (1.1 mg/kg) administered intramuscularly (IM) once daily for five consecutive days. The IM route it ensured consistent and reliable drug delivery without the need for repeated intravenous catheterisation, which would have required additional restraint and carried risks of phlebitis or catheter‐related complications. The IM administration of flunixin meglumine, while not licensed in all countries, is commonly used in equine practice in Brazil. The authors acknowledge that this route may not be standard in all jurisdictions, which limits generalisability.

Firocoxib Group (Gfiro, *n* = 5): Comprised five animals presenting with locomotor problems (e.g., lameness) on clinical examination, with no history of colic, gastrointestinal disease, or NSAID administration in the preceding 6 months. These horses were treated with firocoxib (1.0 mg/kg) administered orally (PO) once daily for five consecutive days. The high dose of firocoxib (1.0 mg/kg) was selected to address a specific knowledge gap in equine pharmacology. The label dose of firocoxib is 0.1 mg/kg for the paste formulation (Equioxx) in many countries. However, clinical scenarios such as severe musculoskeletal pain, acute laminitis, or endotoxemia may prompt clinicians to use higher off‐label doses to achieve more potent analgesia (Davis 2017; Mercer et al. 2023). Despite this common practice, published safety data at supratherapeutic doses are limited (Araújo et al. 2023). The dose of 1.0 mg/kg was chosen as a 10‐fold increase over the label dose to represent a worst‐case supratherapeutic scenario, allowing assessment of the gastric safety margin of COX‐2 selectivity under conditions of high‐dose short‐term use. The authors emphasise that this high dose is off‐label and not recommended for routine clinical use.

Gastroprotective medications were not administered concurrently to any group.

The authors acknowledge important limitations regarding the control group design. First, the control group received IM saline to control for handling and injection stress associated with the flunixin meglumine group. However, the control group did not receive a placebo equivalent for the oral firocoxib administration (e.g., an oral placebo paste). Consequently, the control group does not fully balance the procedural effects of the oral administration route. This means that any stress‐related or handling‐related effects specific to oral dosing are not controlled for in the current design. Second, the control group comprised sound horses, while treatment groups comprised lame horses, introducing selection bias as discussed above. Third, the control group received daily IM injections but no oral placebo, creating differential handling between the control and firocoxib groups. Future studies should employ a double‐dummy design (both IM and oral placebos in control groups) to fully control for route‐specific handling effects. These limitations are further addressed in the discussion.

All horses included in the treatment groups (Gflux and Gfiro) were clinically lame at enrolment (Day 1) and were temporarily removed from mounted patrol duties for the duration of the 5‐day treatment period. These horses remained in their individual pens and received standard care (feeding, watering, and cleaning) but were not exercised or worked during the treatment period. The control group horses (Gcont), being clinically sound, continued their routine mounted patrols of 6 h per day throughout the study period. This difference in activity level between groups represents an additional potential confounder, as exercise and workload may influence gastric mucosal status (Sykes et al. 2019; Van den Boom 2022). The authors acknowledge that this difference limits direct comparison between groups.

Lameness was assessed by a single experienced veterinarian as a binary variable (present or absent) based on clinical examination. Horses were classified as lame if there was an observable alteration in gait or weight‐bearing at walk or trot on a hard surface. No formal lameness grading scale (e.g., AAEP scale) was used. All lame horses (*n* = 10) were assigned to treatment groups (Gflux, *n* = 5; Gfiro, *n* = 5). At the end of the 5‐day treatment period (Day 6), lameness was reassessed using the same binary criteria.

Due to the non‐randomised, descriptive nature of this study, all results should be interpreted as preliminary and hypothesis‐generating. Comparisons between groups are presented for descriptive purposes only and do not imply causal relationships.

### 2.4 Clinical and Behavioural Assessments

A complete anamnesis was performed to record a history of colic, trauma, or NSAID use in the preceding 6 months. This information served dual purposes: (1) to verify compliance with inclusion criteria (absence of recent gastrointestinal disease or NSAID exposure), and (2) to ensure baseline comparability between groups by confirming that no animals had risk factors that could confound interpretation of treatment effects. All enrolled animals met these criteria, and no exclusions were required based on anamnesis findings. Physical examinations, including heart rate, respiratory rate, rectal temperature, capillary refill time, and mucosal colour, were conducted on Day 1 (D1, before treatment) and Day 6 (D6, after treatment). Body condition (BCS) was scored by a single investigator using a scale of 0–5 (Carroll and Huntington 1988). Faecal characteristics (colour, consistency, melena) were evaluated on D1 and D6 (Singer and Smith 2002).

Blood samples were collected from all horses on D1 and D6 via jugular venipuncture using 20‐gauge needles and vacuum tubes. Samples for plasma were collected into tubes containing heparin. Total plasma protein concentration was measured using a refractometer. Plasma fibrinogen concentration was determined using the heat precipitation method (56°C for 3 min) followed by refractometric measurement of precipitated protein. All analyses were performed within 2 h of sample collection by a single trained technician blinded to group allocation.

Behavioural observations were conducted over a 60‐day period prior to the gastroscopic evaluations (Days –60 to –1 relative to Day 1 of the study). Observations were made by a single trained observer who was blinded to the eventual group allocation of the horses. Each horse was observed for a total of 2 h per day, divided into four 30‐min sessions conducted at different times of the day (morning before feeding, morning after feeding, afternoon before exercise, and evening).

In‐stall behaviour (stables), the horses were observed in their individual confined pens (20 m^2^) without human interaction. The observer stood at a distance of approximately 5 m to minimise observer influence. Behaviours recorded included posture (standing, lying sternal, lying lateral), head carriage, ear position, tail position, and interaction with pen mates (through barriers). The horses were observed during routine handling procedures, including haltering, leading from the stall, grooming, and saddling. These observations occurred immediately before the daily mounted patrols. Specific behaviours recorded included resistance to haltering, pulling back on the lead rope, tail swishing, ear pinning, head tossing, biting/kicking attempts, and signs of anxiety (pawing, sweating, defecation/urination during handling).

The horses were also observed during their routine mounted patrols, which consisted of 6 h of active duty per day. Observations focused on behaviour in response to environmental stimuli (traffic, crowds, other horses, loud noises), including startle responses, shying, bolting attempts, refusal to move forward, and signs of fatigue or distress. Working behaviour was evaluated by the same observer accompanying the patrols at a distance of 10–20 m.

Behaviour was evaluated using a standardised ethogram adapted from Arena et al. (2021) and Pessoa et al. (2016), with horses categorised as calm (standing quietly, relaxed ears), nervous (weight shifting, head tossing, tail swishing, flared nostrils, frequent defecation/urination), fearful (avoidance, pulling back, freezing, wide eyes, elevated head carriage, attempting to flee), curious (approaching observer or novel stimuli, ears forward, investigating with muzzle), or aggressive (ears pinned, biting, kicking, charging). For each 30‐min observation session, the predominant behaviour category was recorded, and a horse was assigned to a category if that behaviour was observed for more than 50% of the session duration. Frequencies were calculated across all sessions for each context (stalls, handling/saddling, and policing). For the purpose of comparison between environments (stables vs. policing), behaviours were dichotomised into ‘Quiet’ (calm or curious) and ‘Signs of stress’ (nervous, fearful, or aggressive). This dichotomisation was based on the rationale that both calm and curious behaviours reflect an absence of stress or distress, while nervous, fearful, and aggressive behaviours indicate a stress response (Arena et al. 2021; Pessoa et al. 2016). Table 2 presents the distribution of behaviours between stables and policing environments.

### 2.5 Gastroscopy and Mucosal Evaluation

Gastroscopy was performed on all horses on D1 and D6. The horses fasted for 12 h with water withheld for 3 h prior. Sedation was achieved with intravenous detomidine hydrochloride (0.02–0.04 mg/kg). A 3‐m long, 12 mm diameter flexible videoendoscope was passed through the nostril into the stomach. The squamous and glandular mucosae were systematically inspected, including the fundus, cardia, greater and lesser curvature, *margo plicatus*, antrum, and pylorus. The gastric content was lavage with air and water as needed for visualisation.

Mucosal lesions were identified and graded by a single investigator who was blinded to treatment group allocation. To ensure blinding, all gastroscopy videos and images were coded with animal identification numbers only; group assignment information was withheld from the evaluating investigator until after all grading was completed. Squamous lesions were classified according to the ESGD classification system (Grade 0–IV) (Table 3) (Sykes et al. 2015). Glandular lesions (EGGD) were described by their appearance and contour based on established characterisations (Table 4) (Sykes et al. 2015).

**TABLE 1:** Physiological parameters obtained during the clinical examination on Days 1 (D1) and 6 (D6).

	D1			D6		
Parameters	Gcont (*n* = 10)	Gflux (*n* = 5)	Gfiro (*n* = 5)	Gcont (*n* = 10)	Gflux (*n* = 5)	Gfiro (*n* = 5)
Heart rate (beats/min)	44 ± 5.35	48 ± 13.1	44 ± 11.3	44 ± 2.90	44 ± 2.90	44 ± 12.9
Respiratory rate (breaths/min)	24 ± 3.20	25 ± 6.57	33 ± 9.55	31 ± 17.1	26 ± 4.56	32 ± 4.56
Rectal temperature (°C)	38 ± 1.25	38 ± 0.50	37 ± 0.30	37 ± 0.32	37 ± 0.19	37 ± 0.19
Capillary refill time (seconds)	1.9 ± 1.4	1.6 ± 0.5	1.8 ± 0.4	1.6 ± 0.5	1.6 ± 0.5	1.6 ± 0.5
Mucosal colour (pale, pink, hyperaemic)	Pink	Pink	Pink	Pink	Pink	Pink
Total protein (g/dL)	8.6 ± 1.3	8.0 ± 0.1	8.4 ± 0.4	8.2 ± 0.5	8.6 ± 0.5	8.4 ± 0.6
Fibrinogen (mg/dL)	313 ± 1.44	320 ± 268	280 ± 109	220 ± 109	260 ± 89	260 ± 89

**TABLE 2:** Distribution of behavioural frequencies in stables (in‐stall observation) and during policing activities (mounted patrols).

Behaviour		Stables (in‐stall)	Policing (mounted patrol)
Quiet		9	11
Signs of stress	Nervous	9	5
	Fearful	1	2
	Aggressive	1	2
	Subtotal	11	9
Value of *p* ^a^ (stables vs. policing)		0.6547	0.6547

^a^ Chi‐square test comparing the distribution of behaviours (quiet vs. signs of stress) between stables and policing environments. No statistically significant difference was detected (*p* > 0.05).

### 2.6 Biopsy and Histopathological Analysis

Following endoscopic evaluation, three mucosal biopsy samples were collected from all horses using endoscopic forceps passed through the instrument channel of the endoscope. All biopsies were obtained from the squamous mucosa on the oral (cephalic) side of the *margo plicatus* (the squamous–glandular junction), from the greater curvature region approximately 2 cm oral (cephalic) to the *margo plicatus*.

In horses with visible squamous lesions (ESGD Grade ≥ I), samples were collected from three sites: the centre of the lesion, the periphery of the lesion and an area of endoscopically normal‐appearing mucosa at least 5 cm distant from any lesion.

In horses with no visible lesions (ESGD Grade 0), all three biopsies were obtained from standardised locations along the *margo plicatus* at the 2 o'clock, 4 o'clock, and 8 o'clock positions relative to the cardia, ensuring systematic sampling of the squamous mucosa.

Biopsies were performed immediately following complete gastroscopic examination on both Day 1 (pre‐treatment) and Day 6 (post‐treatment).

The samples were fixed in 10% neutral buffered formalin, processed routinely, embedded in paraffin, sectioned at 4 µm, and stained with haematoxylin and eosin. A single veterinary pathologist, blinded to treatment groups, evaluated all slides. Histological changes were graded using the Equine Gastritis Grading (EGG) system based on the density and distribution of inflammatory cell infiltrate (i.e., infiltration of lymphocytes, plasma cells, macrophages, and neutrophils) and the presence of reactive changes (e.g., hyperkeratosis) (Martineau et al. 2009).

### 2.7 Statistical Analysis

Data were analysed using BioEstat 5.3 software for selected analyses. Descriptive statistics were initially generated for all variables. Categorical data (behaviour, body condition score, fecal characteristics, and ESGD grades) were expressed as absolute frequencies with 95% confidence intervals calculated using the Wilson score method. For continuous clinical parameters (heart rate, respiratory rate, rectal temperature, capillary refill time, total protein, fibrinogen), normality of distribution was assessed using the Shapiro–Wilk test. Variables meeting normality assumptions (Shapiro–Wilk *p* > 0.05) were expressed as mean ± standard deviation. For between‐group comparisons, normally distributed variables were analysed using one‐way ANOVA with Tukey's post hoc test, while non‐normally distributed variables were analysed using the Kruskal–Wallis test with Dunn's post hoc test. For within‐group comparisons between Day 1 and Day 6, paired *t*‐tests were employed for normally distributed continuous data to account for the repeated measures design, with each animal serving as its own control. A priori sample size calculation was not performed due to the pragmatic nature of this clinical study, which utilised a convenience sample.

Throughout this manuscript, statements implying absence of effect should be interpreted as ‘no detectable effect within the limits of this study’. The absence of statistically significant findings does not constitute evidence of no effect, but rather reflects the limited power to detect effects other than very large ones. Within the detectable limits of this small study, no large detrimental effects were detected.

Given the non‐randomised, descriptive nature of this study, all statistical analyses are presented for exploratory purposes only. *p*‐values and confidence intervals are provided to describe the strength of evidence against the null hypothesis of no association within this specific sample, not to infer causality. No adjustments for baseline differences between groups (e.g., the higher prevalence of marked inflammatory cell infiltrate in Gflux at Day 1) are possible given the study design. Results should be interpreted with caution and viewed as hypothesis‐generating.

## 3 Results

### 3.1 Behavioural Assessment

In behavioural assessment, most animals exhibited calm behaviour during mounted patrol work (11/20 horses), while in the stables (in‐stall observation), the majority displayed signs of stress such as nervousness, fearfulness, or aggression (11/20 horses). During handling and saddling, behaviours were predominantly categorised as nervous (8/20 horses) or calm (7/20 horses), with fewer horses showing fearful (3/20) or aggressive (2/20) responses. The comparison distribution of behavioural frequencies during between stables and policing environments is presented in Table 2.

**TABLE 3:** Characterisation of mucosal changes in Equine Gastric Glandular Disease (EGGD) (adapted from Sykes et al. 2015).

ANATOMICAL REGION	CHANGES IN THE GLANDULAR MUCOSA
CARDIA	Flat and haemorrhagic
BOTTOM	Flat and fibrinosuppurative
ANTRUM	Protruding and haemorrhagic Protruding and fibrinosuppurative
PYLORUS	Depressed with/without blood clot Depressed and fibrinosuppurative

### 3.2 Body Condition Score (BCS)

All animals evaluated presented BCS between 3 and 4 (good to fat), indicating adequate nutritional status with well‐covered ribs, no dorsal groove, and barely palpable spinous processes.

### 3.3 Faecal Characteristics

Analysis of faecal samples on days 1 (D1) and 6 (D6) revealed that all animals had faeces of yellowish‐green colour and a semi‐formed to loose, ruminant‐type consistency (similar to cattle feces). These findings were consistent across all groups (Gcont, Gflux, and Gfiro) at both time points. The observed fecal characteristics are not typical for healthy adult horses on a standard diet; normal equine feces should be brown and well‐formed with distinct fecal balls. The alterations observed likely reflect the diet (high concentrate with incorporated alfalfa, lack of pasture access) and the stress of confinement rather than any treatment effect. Importantly, no horse in any group exhibited melena (dark, tarry blood in feces), which would indicate upper gastrointestinal bleeding.

### 3.4 Clinical Examination and Physiological Parameters

No pre‐existing diseases, recent colic episodes, or prior use of NSAID (within 60 days) were reported in the animals' histories. Clinical examinations on D1 and D6 showed no signs of lethargy, anorexia, or abdominal discomfort. There were no significant changes (*p* > 0.05) in heart rate (HR), respiratory rate (RR), rectal temperature (T) or capillary refill time (CRT) between groups or between evaluation days. The mucosal colour was pink in all animals. Total plasma protein and fibrinogen levels also did not show significant alterations. The physiological parameters are detailed in Table 1.

**TABLE 4:** Classification of mucosal changes in Equine Squamous Gastric Disease (ESGD).

GRADE	SQUAMOUS MUCOSA DESCRIPTION
0	The epithelium is intact and there is no appearance of hyperaemia or hyperkeratosis.
I	The epithelium is intact with areas of hyperaemia and hyperkeratosis.
II	Small, single, or multifocal lesions.
III	Large, single, or extensive superficial lesions.
IV	Extensive lesions with areas of deep ulceration.

Review of anamnesis data confirmed that all groups were comparable at baseline with respect to history of colic, trauma, and prior NSAID exposure (none reported in any animal within the preceding 6 months). Physiological parameters (heart rate, respiratory rate, rectal temperature, capillary refill time) were within normal ranges for all horses at both time points and did not differ significantly between groups at baseline (Table 1), suggesting that lame and sound horses were systemically stable at the time of enrolment. However, we acknowledge that subtle differences in stress, pain perception, or subclinical inflammation, not captured by these parameters, may have existed between groups and could represent unmeasured confounders.

It is essential to emphasise that the treatment groups (lame horses) and control group (sound horses) differed fundamentally at baseline. Lame horses may experience chronic pain, stress, and systemic inflammation associated with their underlying musculoskeletal pathology, all of which can independently influence gastric mucosal integrity. Consequently, any observed differences (or lack thereof) may be attributable to these baseline differences in health status rather than to the NSAID treatments themselves. The absence of lesion progression in treated groups compared to controls therefore does not establish safety, as the groups were not comparable at baseline. These baseline differences represent a major confounder that precludes direct causal comparisons between groups, and the results presented should be interpreted as descriptive only.

### 3.5 Response to Drug Therapy

All animals (*n* = 10) with locomotor problems who received 5‐day therapy with flunixin meglumine (Gflux) or firocoxib (Gfiro) showed clinical improvement. Lameness resolved completely in 7 horses (Gflux: 4/5; Gfiro: 3/5) and improved markedly (lameness no longer observable at walk but still subtle at trot) in the remaining 3 horses (Gflux: 1/5; Gfiro: 2/5). No horse in the control group (Gcont) showed lameness at any time point. The use of NSAIDs effectively resolved lameness in all treated horses by Day 6 compared to Day 1, although subtle lameness persisted at trot in 3 horses.

### 3.6 Gastroscopy Findings

Despite a fast of 12 h, a small amount of ingesta was present, but did not exclude mucosal examination. No cases of Equine Gastric Glandular Disease (EGGD) were diagnosed. Only Equine Squamous Gastric Disease (ESGD) was observed, including in the control group (Gcont).

In both D1 and D6, an animal in Gcont presented a squamous mucosal alteration classified as ESGD Grade I, characterised by an intact mucosa with hyperkeratosis, which did not show progression between days. The remaining animals in Gcont had intact mucosa.

In the Gflux group, one horse was diagnosed with ESGD Grade I (extensive hyperkeratosis) on both D1 and D6. In the Gfiro group, one horse was diagnosed with ESGD Grade II (small, single, or multifocal lesions) on both D1 and D6. The alterations found were not related to treatments, as they were present in D1 and did not change significantly after treatment. Biopsy samples were successfully obtained from all 20 horses at both time points (Day 1 and Day 6) without complications. In the 17 horses with ESGD Grade 0 mucosa (9 Gcont, 4 Gflux, 4 Gfiro), biopsies were obtained from standardised locations as per protocol; in the 3 horses with ESGD Grade I‐II lesions (1 Gcont, 1 Gflux, 1 Gfiro), lesion‐targeted sampling was performed as described. The frequency of macroscopic ESGD grades per group is summarised in Table 5.

**TABLE 5:** Frequency and distribution of the grades of Equine Squamous Gastric Disease (ESGD) diagnosed by gastroscopy in control horses treated with NSAID (Day 1) and after (Day 6) therapy.

ESGD grade (Sykes et al. 2015)	Gastroscopy Day 1 (D1)			Gastroscopy Day 6 (D6)		
	Gcont (*n* = 10)	Gflux (*n* = 5)	Gfiro (*n* = 5)	Gcont (*n* = 10)	Gflux (*n* = 5)	Gfiro (*n* = 5)
0	9 (90%)	4 (80%)	4 (80%)	9 (90%)	4 (80%)	4 (80%)
I	1 (10%)	1 (20%)	0 (0%)	1 (10%)	1 (20%)	0 (0%)
II	0 (0%)	0 (0%)	1(20%)	0 (0%)	0 (0%)	1 (20%)
III	0 (0%)	0 (0%)	0 (0%)	0 (0%)	0 (0%)	0 (0%)
IV	0 (0%)	0 (0%)	0 (0%)	0 (0%)	0 (0%)	0 (0%)
p‐value^a^ (vs. Gcont at D1/D6)	−	1.000	0.455	−	1.000	0.455
p‐value^b^ (D1 vs. D6 within‐group)	1.000	1.000	1.000	−	−	−

^a^ No statistically significant differences were found (*p* > 0.05). Within‐group comparisons with Fisher's exact test between D1 and D6 for all groups also showed no significant changes in ESGD grade distribution.

^b^ Within‐group comparison between Day 1 and Day 6: McNemar's test for paired nominal data (dichotomised as Grade 0 vs. Grade ≥ I). No statistically significant changes were detected in any group.

Figure 1 illustrates the normal gastric mucosa (A) and the alterations found: hyperkeratosis in Gcont (B), slight hyperaemia in Gfiro (C) and extensive hyperkeratosis in Gflux (D). All diagnosed lesions were located in the squamous mucosa near the *margo plicatus*.

**FIGURE 1:**
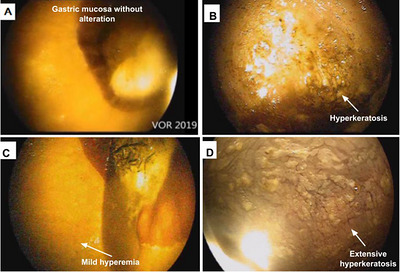
Demonstrates the normal equine stomach mucosa and alterations found in the squamous mucosa. (A) Demonstrates the intact gastric mucosa of a horse from Gcont, squamous mucosa with a lighter colouration, and glandular mucosa with a darker colouration. (B) Hyperkeratosis diagnosed in an animal from Gcont. (C) Small focal lesion (ESGD Grade II) diagnosed in a Gfiro animal. (D) Extensive hyperkeratosis diagnosed in a Gflux animal.

Due to the small sample size and consequent low statistical power, the absence of detectable change should not be interpreted as evidence of no effect. Under these specific conditions (5‐day treatment in mature, clinically stable police horses with low baseline lesion prevalence), no detectable increase in ESGD grade was observed. Examination of individual animal data confirmed that no horse in any group experienced progression or regression of lesion grade between the two time points, indicating stability of the squamous mucosa throughout the study period regardless of treatment.

### 3.7 Histopathological Findings

Histological changes, classified according to the Equine Gastritis Grading System (EGG), are presented in Table 6.

**TABLE 6:** Histological grading of gastric squamous mucosa according to the Equine Gastritis Grading (EGG) system before (Day 1) and after (Day 6) NSAID therapy.

EGG grading (Martineau et al. 2009)	Gastroscopy Day 1 (D1)			Gastroscopy Day 6 (D6)		
Inflammatory cell infiltrate density, n (%)	Gcont (*n* = 10)	Gflux (*n* = 5)	Gfiro (*n* = 5)	Gcont (*n* = 10)	Gflux (*n* = 5)	Gfiro (*n* = 5)
0 (Normal)	2 (20%)	2 (40%)	2 (40%)	2 (20%)	3 (60%)	2 (40%)
1 (Mild)	7 (70%)	0 (0%)	2 (40%)	6 (60%)	0 (0%)	2 (40%)
2 (Moderate)	1 (10%)	0 (0%)	1 (20%)	2 (20%)	2 (40%)	2 (40%)
3 (Marked)	0 (0%)	3 (60%)	0 (0%)	0 (0%)	0 (0%)	0 (0%)
Hyperkeratosis	1 (10%)	1 (20%)	0 (0%)	1 (10%)	1 (20%)	0 (0%)
p‐value^a^		1.000	1.000		1.000	1.000

*Note*: Biopsy samples were obtained from standardised locations adjacent to the *margo plicatus* in all horses, with additional lesion‐targeted sampling in the three horses with macroscopic ESGD Grade ≥ I. All samples were collected immediately following gastroscopic examination on each evaluation day.

^a^ Between‐group comparison to mucosal lesion progression: Fisher's exact test comparing each treatment group to control (Gcont). No significant differences were detected.

At Day 1 (D1): The density of the inflammatory cell infiltrate varied across groups. In Gcont, most animals showed mild infiltrate (7/10, 70%), with one animal (10%) showing moderate infiltrate. In Gflux, three animals (60%) presented with marked infiltrate (Grade 3), while no animals in Gcont or Gfiro exhibited marked infiltrate at baseline. In Gfiro, two animals (40%) had normal infiltrate, two (40%) mild, and one (20%) moderate.

At Day 6 (D6): Following the 5‐day treatment period, changes in inflammatory cell infiltrate density were observed. In Gcont, six animals (60%) continued to show mild infiltrate, while moderate infiltrate increased from one to two animals (20%). In Gflux, the marked infiltrate present in three animals at D1 resolved completely by D6; instead, two animals (40%) showed moderate infiltrate and three (60%) had normal infiltrate. In Gfiro, two animals (40%) remained normal, two (40%) mild, and two (40%) moderate, representing a slight increase in moderate infiltrate compared to D1 (one animal).

All inflammatory cell infiltrates were focal in distribution (Figure 2). Hyperkeratosis was the only reactive change observed in the squamous mucosa. At D1, hyperkeratosis was present in one animal in Gcont (10%) and one animal in Gflux (20%). At D6, hyperkeratosis persisted in the same Gcont animal (10%) and the same Gflux animal (20%). No hyperkeratosis was observed in Gfiro at either time point.

**FIGURE 2:**
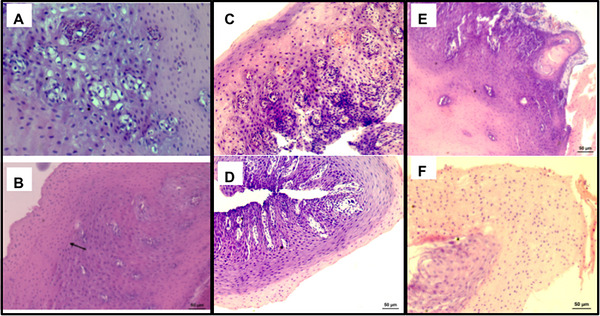
Histopathological alterations of the squamous mucosa of the stomach diagnosed in Gcont, Gflux, and Gfiro. (A) Hydropic degeneration with moderate cellular infiltrates in Gcont (D1). (B) Hyperkeratosis in the squamous mucosa in Gcont (D1). (C) Discrete area with hydropic degeneration and cellular infiltrate with moderate density in Gflux (D1). (D) Hyperkeratosis with thickening of the stratum corneum with moderate cellular infiltrate in Gflux (D6). (E) Hyperkeratosis with moderate density infiltrate in Gfiro (D1). (F) Hyperkeratosis with moderate density infiltrate in Gfiro (D6).

Given the limited statistical power of this study (powered only to detect very large effects), these non‐significant *p*‐values should not be interpreted as evidence of stability or absence of change. Under these specific conditions, no detectable increase in histopathological scores was observed. However, the wide confidence intervals and low power mean that smaller but potentially clinically relevant changes cannot be excluded. The risk of Type II error is substantial.

## 4 Discussion

Non‐steroidal anti‐inflammatory drugs remain an essential component of the therapeutic management of horses for conditions ranging from colic to musculoskeletal pain. However, their use is tempered by the well‐documented risk of gastrointestinal toxicity, a significant component of Equine Gastric Ulcer Syndrome (EGUS) (Flood and Stewart 2022; Vokes et al. 2023). The clinical dilemma often involves balancing potent anti‐inflammatory and analgesic effects with mucosal safety. This study provides preliminary descriptive data from blinded gastroscopic and histopathological evaluation suggesting that, within the limitations of a small non‐randomised design, label‐dose flunixin meglumine (1.1 mg/kg IM) or a high dose of firocoxib (1.0 mg/kg PO) administered for 5‐day was not associated with detectable increases in gastric mucosal injury of adult horses. The findings offer preliminary descriptive data that may inform clinical reasoning, but given the methodological limitations, they should not be used as definitive guidance for NSAID selection. Practitioners must interpret these results with full awareness of the study's constraints and the need for confirmatory evidence.

Non‐steroidal anti‐inflammatory drugs induce gastric mucosal injury primarily through inhibition of cyclooxygenase (COX) enzymes, which catalyse the conversion of arachidonic acid to prostaglandins (Cook and Blikslager 2015; Flood and Stewart 2022). Two COX isoforms exist: COX‐1 is expressed in most tissues, including the gastric mucosa, where it synthesises prostaglandins (primarily PGE2 and PGI2) that mediate gastroprotective effects such as stimulation of mucus and bicarbonate secretion, maintenance of mucosal blood flow, and promotion of epithelial repair. COX‐2 is inducible and primarily associated with inflammation and pain (Cook and Blikslager 2015).

Non‐selective NSAIDs such as flunixin meglumine inhibit both COX‐1 and COX‐2, thereby reducing gastroprotective prostaglandins while providing anti‐inflammatory effects. This dual inhibition accounts for their therapeutic efficacy but also their gastrointestinal toxicity. In contrast, COX‐2 selective NSAIDs like firocoxib are designed to spare COX‐1, theoretically preserving gastroprotective prostaglandin synthesis while still inhibiting the inflammatory cascade (Cook and Blikslager 2015; Stiller and Hjemdahl 2022).

Additional mechanisms may contribute to NSAID‐induced gastric injury, including topical irritation (particularly with oral administration), induction of leukocyte adhesion to the gastric microvasculature, and reduction of growth factor expression (Camacho‐Luna et al. 2018; Flood and Stewart 2022). The squamous mucosa of the equine stomach is particularly vulnerable to NSAID injury as it lacks the protective mucus and bicarbonate barrier present in the glandular mucosa (Van den Boom 2022). The present study did not find detectable increases in squamous lesions following short‐term administration of label‐dose flunixin meglumine or high‐dose firocoxib, though the small sample size and low power preclude definitive conclusions.

Previous studies have compared the gastric effects of various NSAIDs in horses, though none have specifically examined label‐dose flunixin meglumine versus high‐dose firocoxib using serial gastroscopy. Comparative investigations between flunixin meglumine and firocoxib have primarily focused on clinical efficacy, cyclooxygenase inhibition, or specific outcomes such as post‐operative recovery and endotoxemia management, rather than direct, blinded gastroscopic evaluations of gastric mucosa (Cook et al. 2009; Duz et al. 2015; Ziegler et al. 2019). Existing safety assessments have typically utilised standard label doses, leaving a gap in knowledge regarding the gastric effects of supratherapeutic dosing, which may occur in clinical practice (Araújo et al. 2023; Bishop et al. 2023). The present study addresses this gap by evaluating a 10‐fold increase over the label dose of firocoxib (1.0 mg/kg) in direct comparison with label‐dose flunixin meglumine (1.1 mg/kg). Our findings provide descriptive data suggesting that, under the specific conditions examined, high‐dose firocoxib was not associated with detectable gastric injury within the limits of this small pilot study. However, direct comparisons with previous work are constrained by differences in study design, sample size, dosing regimens, and outcome measures. The present results should be considered hypothesis‐generating and interpreted alongside the larger body of evidence with appropriate caution (Camacho‐Luna et al. 2018; Sykes et al. 2019; Van den Boom 2022).

The findings of the present study can be contextualised by comparison with earlier investigations of NSAID effects on the equine gastric mucosa. MacAllister et al. (1993) compared the adverse effects of phenylbutazone (4.4 mg/kg PO q12h), flunixin meglumine (1.1 mg/kg IV q24h), and ketoprofen (2.2 mg/kg IV q24h) administered for 7–10 days in horses. They reported that all three NSAIDs induced some degree of gastric mucosal injury, with phenylbutazone associated with the most severe lesions (gastric ulcer scores of 2–3 on a 0–4 scale), while flunixin meglumine and ketoprofen produced milder lesions (scores of 1–2). Notably, the study by MacAllister et al. used intravenous rather than intramuscular administration of flunixin meglumine, and the treatment duration was longer (7–10 days vs. 5 days in the present study). Despite these differences, both studies found that flunixin meglumine was associated with relatively mild gastric injury compared to phenylbutazone.

More recently, Richardson et al. (Richardson et al. 2018) compared the effects of selective (firocoxib, 0.1 mg/kg PO q24h) versus nonselective (flunixin meglumine, 1.1 mg/kg IV q12h) COX inhibitors on gastric ulceration scores and intestinal inflammation in horses undergoing surgery. They reported that flunixin meglumine was associated with higher gastric ulcer scores compared to firocoxib, supporting the concept that COX‐2 selective NSAIDs may have a superior gastric safety profile. However, that study evaluated label‐dose firocoxib (0.1 mg/kg), whereas the present study used a 10‐fold higher dose (1.0 mg/kg). The absence of detectable gastric injury with high‐dose firocoxib in the present study extends the findings of Richardson et al. by suggesting that even at supratherapeutic doses, firocoxib may not induce detectable squamous lesions under short‐term administration. However, direct comparisons are constrained by differences in study design (surgical model vs. lameness model), sample size, and outcome measures, and the present results should be considered hypothesis‐generating. Flunixin meglumine has a half‐life of approximately 1.5–2 h in horses, necessitating administration every 12 h (q12h) for sustained analgesia in many regions, although once‐daily (q24h) dosing is also practiced in some countries, including Brazil (Duz et al. 2019; Mercer et al. 2023). The present study used q24h intramuscular administration (1.1 mg/kg) based on local clinical practice, which may have influenced the gastric findings. Compared to q12h dosing, q24h administration results in lower peak plasma concentrations but longer intervals without therapeutic drug levels, potentially reducing both anti‐inflammatory efficacy and gastrointestinal toxicity, and the total daily dose (1.1 mg/kg) is half that of q12h regimens (2.2 mg/kg/day) (Duz et al. 2019; Mercer et al. 2023). Therefore, the absence of detectable gastric injury in the flunixin meglumine group may be attributable to the lower total daily dose rather than to inherent drug safety, and the results should not be interpreted as evidence that flunixin meglumine is free of gastric risk at higher total daily doses. Future studies directly comparing q12h and q24h regimens are needed to address this question.

Intramuscular administration of flunixin meglumine, while used in some regions for convenience, is associated with potential adverse effects that should be acknowledged. These include local injection site reactions (swelling, pain, and fibrosis), muscle damage (elevated creatine kinase and aspartate aminotransferase), and the potential for inadvertent intra‐arterial injection, which can lead to severe complications such as seizures, ataxia, or recumbency (Davis 2017; Mercer et al. 2023). The intramuscular route was selected for this study for pragmatic reasons (consistent drug delivery, avoidance of repeated intravenous catheterisation, and direct comparison with the IM saline control group). The authors emphasise that intramuscular administration is not licensed for flunixin meglumine in all countries, and the intravenous route remains the standard of care in many jurisdictions. Clinicians should consult local regulations and product labelling when selecting the route of administration.

The present study evaluated the gastric effects of high‐dose firocoxib (1.0 mg/kg, a 10‐fold increase over the label dose) but did not assess renal function. This represents an important limitation, as COX‐2 selective NSAIDs, including firocoxib, can affect renal function by inhibiting prostaglandin‐mediated renal blood flow regulation, particularly in volume‐depleted or hypotensive patients (Cook and Blikslager 2015; Davis 2017; Mercer et al. 2023). Prostaglandins synthesised by COX‐2 play a critical role in maintaining renal perfusion under conditions of reduced blood flow. High‐dose COX‐2 inhibition could therefore theoretically increase the risk of acute kidney injury, especially in dehydrated horses or those with pre‐existing renal disease. The 1.0 mg/kg dose used in this study is off‐label, and the safety of this dose with respect to renal function has not been established. Clinicians considering high‐dose firocoxib should monitor renal parameters (serum creatinine, urea, and urine output) and ensure adequate hydration. Future studies should include renal function assessment when evaluating high‐dose firocoxib.

The results align with and extend the findings of several recent investigations into the comparative safety of these NSAIDs. Studies have focused primarily on clinical results, cyclooxygenase inhibition, or specific models of intestinal injury (Cook et al. 2009; Duz et al. 2015; Ziegler et al. 2019). Our study extends this work by applying the gold‐standard diagnostic tool, gastroscopy, to visualise gastric mucosal changes. Given the small sample size and consequent low statistical power, the absence of significant worsening of ESGD grades or histopathological scores in treated groups compared to controls indicates that, within the limits of this small study, no large detrimental effects were detected. However, due to the power limitations, this finding should not be interpreted as evidence that high‐dose firocoxib is free of gastric risk. The confidence intervals for lesion prevalence are wide and include the possibility of increases in injury that this study was underpowered to detect. This is consistent with the work of Bishop et al. (2023), who found no significant effect of label dose firocoxib or flunixin meglumine on the thickness of the large colon mural, another indicator of gastrointestinal health. Furthermore, the present study contributes descriptive endoscopic data to the safety assessment of firocoxib, which has been advocated for its potential gastro‐sparing effects. Given the substantial methodological limitations (non‐randomised design, small sample size, low statistical power, and baseline group incomparability), the present study should be considered hypothesis‐generating (Cook and Blikslager 2015).

In particular, the context, using a high dose of firocoxib, addresses a specific knowledge gap in equine pharmacology. Although label doses are established for anti‐inflammatory effects, clinical scenarios such as severe pain or endotoxemia can prompt clinicians to use higher doses (Davis 2017; Mercer et al. 2023). Previous safety assessments, such as that of Araújo et al. (2023), have typically evaluated therapeutic doses. Our findings are consistent with the possibility that, under these specific conditions, a short‐term increase to 1.0 mg/kg PO daily does not cause acute, severe gastric mucosal damage of a magnitude detectable with this sample size. However, more modest but still clinically relevant injury cannot be excluded. The point estimates for lesion prevalence in treatment groups (Gflux: 80% Grade 0 at D1 and D6; Gfiro: 80% Grade 0 at D1 and D6) were similar to those in the control group (Gcont: 90% Grade 0 at D1 and D6). However, as shown in Table 5, the 95% confidence intervals for these proportions are wide due to the small sample size. For example, the confidence interval for the control group (90% [55–100%]) and for the firocoxib group (80% [28–99%]) substantially overlaps. This overlap means that the observed data are compatible with a true lesion prevalence in the treatment groups that is lower than, equal to, or higher than that in the control group. Consequently, the confidence intervals are consistent with both no effect and an increase in risk. The wide intervals reflect the imprecision inherent in small‐sample studies and underscore that no definitive conclusions about safety or risk can be drawn.

The isolated squamous mucosal lesions (Grade I‐II ESGD) observed in individual horses in all groups, including controls, underscore the multifactorial nature of EGUS. These pre‐existing lesions did not show progression, indicating that the brief NSAID regimen was not a primary aggravating factor. This highlights the importance of baseline gastroscopy in clinical studies and the reality that many performance and confined horses have subclinical gastric disease irrespective of NSAID use (Van den Boom 2022). It reinforces the consensus that factors such as management, diet, and stress play a crucial role in the pathogenesis of squamous disease, while short‐term pharmacologic insult can be less consequential than previously feared (Sykes et al. 2019).

The behavioural assessment served to contextualise the gastric findings, given that chronic stress is a known risk factor for EGUS through activation of the hypothalamic–pituitary–adrenal axis and increased cortisol production, which can compromise gastric mucosal defence mechanisms (Sykes et al. 2019; Van den Boom 2022). Baseline behavioural profiles revealed comparable stress‐related behaviours between lame and sound horses across both stable and working environments, supporting that stress levels were similar between groups prior to treatment. This comparability helps interpret the absence of lesion progression, as it reduces, though does not eliminate, the possibility that stress, rather than NSAID treatment, drove any observed differences in gastric outcomes. The horses were active police animals routinely exposed to urban stressors such as traffic, crowds, and loud noises, with behavioural responses representative of working horses in such environments (Arena et al. 2021; Pessoa et al. 2016). Accordingly, findings may not generalise to horses kept under different management conditions or with distinct behavioural profiles. Future studies should incorporate physiological stress measures, such as fecal cortisol metabolites or heart rate variability, to complement behavioural observations.

The management system employed in this study, long‐term confinement without pasture access and reliance on a concentrate‐based diet with incorporated forage, warrants consideration when interpreting the gastric findings. Although this regimen reflects the reality of many police and competition horses (Pessoa et al. 2016), management under this regime can interfere with gastric health and animal welfare (Van den Boom 2022). The presence of low‐grade lesions (ESGD Grade I‐II) in all groups, including controls, likely reflects this management background rather than acute NSAID effects. Importantly, short‐term NSAID administration did not exacerbate pre‐existing lesions, as evidenced by the absence of lesion progression in all groups.

However, this study has important limitations that must be considered when extrapolating the results. The sample size was small (*n* = 5 per treatment group), limiting statistical power to detect subtle or less frequent mucosal changes. The duration of treatment was brief (5 days); the safety profile of prolonged high‐dose administration remains unknown and can differ significantly as cumulative effects manifest (Spanton 2024). The study population consisted of mature, clinically stable police horses; results may not be translated to younger, older, critically ill, or dehydrated patients in whom the pharmacokinetics and toxicodynamics of NSAID are altered (Camacho‐Luna et al. 2018). Finally, the evaluation was limited to the stomach; NSAID‐associated right dorsal colitis or other distal intestinal effects were not assessed (Camacho‐Luna et al. 2018).

The non‐randomised group allocation, assigning lame horses to treatment groups and sound horses to controls, represents a major source of selection bias that fundamentally limits the interpretability of the study. The treatment groups (lame horses) and control group (sound horses) differed not only in NSAID exposure but also in pain status, stress levels, systemic inflammation, activity level, and potentially other unmeasured variables. These differences cannot be statistically adjusted for given the study design. While baseline anamnesis and clinical data confirmed comparability in terms of historical risk factors for EGUS, the presence of lameness itself introduces confounding that cannot be disentangled from treatment effects. No causal conclusions can be drawn.

The small sample size is a limitation that constrains the conclusions that can be drawn from this study. The study was adequately powered only to detect very large effects (e.g., a 50% increase in lesion prevalence). This limitation is particularly important given the low baseline prevalence of lesions in this population (10%), which further reduces statistical power to detect treatment‐associated increases. The non‐significant findings reported in this study should therefore not be interpreted as evidence of safety, but rather as an absence of detectable large effects under the specific conditions studied. Adequately powered studies to detect moderate effect sizes would require larger sample sizes.

A fundamental limitation must be emphasised at the outset: due to the non‐randomised allocation design, no causal conclusions can be drawn from this study. The assignment of lame horses to treatment groups and sound horses to the control group introduces systematic bias, as pain, stress, and underlying pathology may influence the gastric mucosa independently of treatment. This study is therefore best characterised as a descriptive pilot study providing hypothesis‐generating data rather than confirmatory evidence. Readers should not interpret any comparison between groups as indicative of a causal treatment effect. The analyses presented are descriptive and exploratory; statistical tests are used to summarise patterns in the data rather than to test causal hypotheses.

Several additional limitations warrant discussion. First, the intramuscular route used for flunixin meglumine, while pragmatic for this study, is not licensed in all countries and may not reflect standard clinical practice in some jurisdictions. This limits the generalisability of findings related to flunixin meglumine. Second, the high dose of firocoxib (1.0 mg/kg) represents a 10‐fold increase over the label dose and is off‐label. While this dose was chosen to explore the safety margin under worst‐case conditions, it does not reflect routine clinical use, and the findings should not be interpreted as supporting high‐dose firocoxib in practice without further safety data. Third, and most critically, the control group design was imbalanced: the control group received IM saline (flunixin group) but no oral placebo (firocoxib group). This means that procedural stress specific to oral administration was not accounted for in the control group. While the absence of detectable gastric injury in the firocoxib group suggests that any such unaccounted procedural effects were minimal, this imbalance remains a methodological limitation. Future studies should employ a double‐dummy design (Stiller and Hjemdahl 2022) in which control animals receive both IM and oral placebos to fully balance handling across all routes of administration.

Future studies should build on these findings by investigating longer durations of treatment (e.g., 2–4 weeks) with standard and high doses of firocoxib and flunixin meglumine in larger, more diverse equine populations. Incorporating additional biomarkers of gastrointestinal inflammation, such as faecal myeloperoxidase or serum gastrin, could provide more sensitive indicators of subclinical injury (Frippiat et al. 2025). Comparative studies in horses with pre‐existing EGUS would be highly valuable to assess the risk of exacerbation. Furthermore, pharmacokinetic–pharmacodynamic modelling could help define the optimal dose of firocoxib that maximises analgesia while minimising gastrointestinal risk in different clinical syndromes.

This descriptive pilot study provides preliminary endoscopic and histopathological data indicating that, under the specific conditions examined (5‐day treatment in mature, clinically stable police horses with low baseline lesion prevalence), no detectable increase in gastric mucosal lesions was observed beyond that seen in untreated controls. However, due to the non‐randomised design, small sample size, and limited statistical power, this finding should not be interpreted as evidence that high‐dose firocoxib does not induce gastric mucosal injury. Rather, it indicates that no large‐magnitude increase was detectable within the limits of this study. The results are hypothesis‐generating and require confirmation in adequately powered randomised trials before any clinical recommendations can be made.

## 5 Conclusion

This study provides preliminary, hypothesis‐generating data on the gastric effects of short‐term high‐dose firocoxib (1.0 mg/kg) compared to label‐dose flunixin meglumine (1.1 mg/kg) in a small select group of horses under specific management conditions. Due to the non‐randomised allocation design (lame horses in treatment groups, sound horses as controls), no causal conclusions can be drawn regarding treatment effects on gastric mucosa. Given the small sample size and consequent low statistical power, the absence of detectable increase should not be interpreted as evidence of safety or as proof that high‐dose firocoxib does not induce gastric injury. These findings are hypothesis‐generating and require confirmation in adequately powered randomised trials.

## Author Contributions

**Renato A. de Oliveira**: conceptualisation, funding acquisition, writing – original draft, investigation, data curation, formal analysis. **Alexandre da S. Correa**: conceptualisation, funding acquisition, writing – review and editing, methodology, project administration, supervision, resources, formal analysis. **Francisco Décio de O Monteiro**: conceptualisation, writing – review and editing, methodology, supervision, resources, formal analysis. **Pedro Paulo M. Teixeira**: conceptualisation, writing – review and editing, methodology, supervision, resources, formal analysis.

## Funding

The authors have nothing to report.

## Ethics Statement

The study was conducted in accordance with the recommendations of the Brazilian National Council for the Control of Animal Experimentation (CONCEA) and was approved by the Ethics Committee on the Use of Animals of the Federal University of Pará (UFPA) under protocol number 8805210920. This prospective, controlled clinical study was performed on a population of horses of the military police battalion.

## Conflicts of Interest

The authors declare that they have no competing interests.

## Data Integrity Statement

Francisco Décio de Oliveira Monteiro had full access to all the data in the study and takes responsibility for the integrity of the data and the accuracy of data analysis.

## Clinical Study Registration Number

As a veterinary study with a small sample size and no clinical trial registration requirement under applicable guidelines, this study was not registered in a public trials registry. All procedures were approved by the institutional animal ethics committee (protocol 8805210920, approved 15 March 2019) prior to commencement.

## Data Availability

The data that support the findings of this study are available from the corresponding author upon reasonable request.
